# A Blueprint for National and International Healthcare Data Ecosystems: Learning From Building STRONG‐AYA

**DOI:** 10.1002/lrh2.70132

**Published:** 2026-11-08

**Authors:** Dan Stark, Oana Lindner, Simone Hanebaum, Nicola Hughes, Urska Kosir, Winette Van Der Graaf, the Members of the Strong‐AYA Consortium, Julien Bollard, Helen Boyle, Mehdi Brahmi, Alizée Diatchenko, Thierry Durand, Perrine Marec‐Berard, Christèle Riberon, Clemence Romeo, Eric Bienefeld, Norbert Couespel, Alberto Hermosel, Jack Latteur, Nela Osmanovic, Richard Price, Juliana de Sa, Eduardo Veber, Paolo Baili, Simone Bonfarnuzzo, Andrea Ferrari, Chiara Prampolini, Salvatore Provenzano, Annalisa Trama, Florin Barnea, Mariana Coutinho, Urska Kosir, Katalin Rizvine Lehoczky, Marc Deloger, Nathalie Gaspar, Lucy Metayer, Siham Moulai, Benjamin Verret, Bart van Beusekom, Gijs Geleijnse, Faatimah Patel, Anna Dąbrowska‐Iwanicka, Paulina Jagodzinska‐Mucha, Agnieszka Janowska, Iwona Lugowska, Ewa Paszkiewicz‐Kozik, Katarzyna Pogoda, Magdalena Rosinska, Piotr Rutkowski, Anna Sikora‐Koperska, Pawel Wiechno, Ananya Choudhury, Andre Dekker, Luis Sanchez Gomez, Varsha Gouthamchand, Joshi Hogenboom, Marine Jacquemin, Flora Lysen, Darian Meacham, David Townend, Leonard Wee, Simone Hanebaum, Maike Hentges, Olga Husson, Silvie Janssen, Winette van der Graaf, Matthew Barker‐Hewitt, Rob Carter, Emily Connearn, Richard Feltbower, Nicola Hughes, Oana Lindner, Lee Norman, Daniel Stark, Charlotte Heaven, Martin McCabe, Danielle Owen, Gareth Price, Kate Vaughan, Charlotte Cairns, Nicole Collaco, Anne‐Sophie Darlington, Katherine Hunt, Samantha Sodergren, Richard Wagland, Kirsty Way, Olga Husson, Leonard Wee

**Affiliations:** ^1^ Division of Oncology Leeds Institute of Medical Research, School of Medicine, University of Leeds Leeds UK; ^2^ Netherlands Cancer Institute Amsterdam the Netherlands; ^3^ Youth Cancer Europe—Youth Changing Europe—Youth Cancer Europe Cluj‐Napoca Romania; ^4^ University of Maastricht Maastricht the Netherlands

**Keywords:** adolescence period, blueprint, digital health, health care, healthcare system, knowledge management, organizational learning, young adult

## Abstract

**Introduction:**

Healthcare systems aim for continuous improvement to ensure excellent clinical outcomes, including optimized quality of life. This relies on accurate data and evaluation patient‐centered as well as biomedical outcomes. This data increasingly arises from real‐world data (RWD). We are using this data across a multi‐national European consortium, to create a Learning Healthcare System, improve care, policy, and ultimately patients’ outcomes.

**Methods:**

STRONG‐AYA is an interdisciplinary community, led from the Netherlands Cancer Institute, working across frequent consensus meetings, surveys, questionnaire design, data collection, synthesis and privacy‐preserving federated analysis (in Vantage6), to ethically study and apply clinical and patient‐centered outcomes data to learn about healthcare systems for Adolescents and Young Adults (AYAs) diagnosed with cancer when aged 15–39. The young people themselves are at the heart of our decision‐making, and we work to make our data analyses transparent.

**Results:**

A harmonized ‘Core Outcome Set’ was agreed with all stakeholders. We developed definitions and data science methodological approaches, aligned with Learning Healthcare System practices. We have built AYA cancer data ecosystems, at local, national and European levels, to combine informatics with lived experience, clinical and scientific interpretation, based upon initial ‘use cases’ of the mental health consequences and cancer survival rates. We are applying those insights in real time individual clinical care, individual patient education and support as well as service level metrics.

**Conclusions:**

The Strong‐AYA Learning Healthcare System for AYA‐onset cancer can empower discovery by health researchers, digital innovation, and innovative insights for patients, clinicians, managers and policymakers who aim for transformational improvements in the quality and outcomes of current clinical practice though the development of a Learning Healthcare System in AYA‐onset cancer care. Sustainability is our next major challenge.

## 1 Introduction

Healthcare systems aim for continuous improvement to ensure excellent clinical outcomes, including optimized quality of life. This relies on the accurate recording, monitoring, and evaluation of indicators (such as clinical effectiveness, patient safety, patient‐centered, and patient‐reported outcomes—“PROs”). This data increasingly arises from real‐world data (RWD) collection and can then be utilized within “Learning Healthcare Systems,” as evidence for care transformation.

Adolescents and Young Adults (AYAs) have rare cancer types, such as sarcomas, lymphomas, and germ cell malignancies, or uncommon presentations of common cancers for example, early‐onset carcinomas. Generating practice‐changing evidence is challenging, because indicators of care quality and PROs have been (up to now) collected non‐systematically.

### 1.1 AYAs Specific Cancer Care Needs

The existing European gold standard defines “AYA” as people receiving a cancer diagnosis between 15 and 39 years of age inclusive. Worldwide, an estimated 1.2 million AYAs [1] were diagnosed with cancer in 2020; around 5% of all cancer diagnoses [2]. This incidence is increasing [3]. AYA‐specific diagnosis and treatment have recently improved, through focussed specific innovations and research. In high‐income countries over 85% of AYAs with cancer will survive ≥ 5 years [4, 5]. Many live > 50 years beyond cancer treatment [6] as cancer survivors, with increased risks of physical, psychological, and social effects [7, 8, 9], long recognized as specific to the AYA age range [10].

Across many countries cancer treatment systems possess limited knowledge about the complex issue of AYAs' specific needs [11]. AYA cancer is often cared for by either pediatric (< 15–18 years) or adult (> 16) specialists, when AYAs are liminal for both those services (caring mainly for patients aged under 12 or over 70 respectively). AYAs' care requires a specific focus given their priority challenges [1, 2, 12, 13, 14]:

1. *Unique epidemiology:* AYAs develop both pediatric‐ and adult‐type tumors, requiring expertise from both clinical services [15].
2. *Distinct tumor biology:* Cancers in AYAs often exhibit significant age‐specific molecular features and pharmacological responses [15].
3. *Delayed diagnosis leading to more advanced disease:* From a lack of awareness and silos in services [16, 17].
4. *Limited access to clinical trials:* Resulting in slow improvement in outcomes and a reliance on real world data [18].
5. *Personal development:* AYAs are undergoing life transitions (e.g., identity formation, autonomy, and career development) making the balance between best cancer treatments and quality of life difficult to achieve [12, 19].
6. *Survival rate disparities:* Survival improvements for AYAs lag behind those seen in other age groups [20].

### 1.2 The Requirement for AYA‐Specific Cancer Outcomes Data

AYAs with cancer struggle to find care for cancer adapted to their age [21]. Recently, AYA cancer services in some parts of the world have been accepted as distinct, embedding AYA‐specific cancer care [13]. Maintaining this recent momentum requires ongoing Learning Healthcare Systems to collate data and improve outcomes. By learning from routinely and newly collected electronic data on AYA cancer patient care and outcomes, STRONG‐AYA is building a Learning Healthcare System to inform patient, service provider and service‐level decision‐making and policies [22].

The National Cancer Institute (NCI) suggested AYA cancer service providers pool data across institutions and countries; for research [11, 21], to address widespread geographic variation in AYA cancer outcomes, policy and care [13]. Data collected is sometimes “pooled” into large registers, but international differences in research governance and interpretations of patient privacy protection laws limit its use for real world service provision. Novel approaches to enable large‐scale, better powered evidence‐based insights into outcomes are needed to complement traditional data centralization. Cancer outcomes data in AYA is currently not standardized and while commonly used endpoints (such as five‐year overall survival) are important, they do not reflect AYAs' specific needs.

## 2 The STRONG‐AYA Initiative

STRONG‐AYA was created in 2022 to align research methods, clinical research questions, and outcome measurements (including AYA‐specific PROs) [23]. STRONG‐AYA is an interdisciplinary consortium, funded by EU Horizon Europe, of leading patient advocacy organizations, healthcare providers and clinical academic research centers in AYA cancer. Results arising from this work may inform national and European clinical decision‐making, service design, and policy. Through this initiative we are addressing three concrete objectives.

First, the STRONG‐AYA consortium has developed a standardized Core Outcome measurement Set (“COS”) [24], including routinely collected clinical data and PROs, to facilitate the recording, monitoring, and prioritization of outcomes of direct relevance to AYA. The COS is in the process of being implemented across consortium nations (Netherlands, France, Italy, Poland, UK) to generate and use the resulting insights in multiple clinical practices.

Second, we have set up a federated data ecosystems of dedicated skilled people, clinical informatics, and data management, supported by an ethical‐legal governance framework. STRONG‐AYA chose to create five national data ecosystems that collect, collate and utilize clinical and PROs data in AYA cancer service provision, and connect the 5 to create an international (Pan‐European) data ecosystem.

Third, we have implemented a secure federated data analytics infrastructure that enables large‐scale data analyses over multiple independent data repositories, without sharing any individual subject‐level data, in a timely and transparent fashion. This ensures that results of analyses are the same as if all the data had been centralized in a single repository. These analyses will examine within and between‐patient variation, across time and AYA subgroups and settings.

### 2.1 Definition of a Data Ecosystem in Cancer Care

Our data ecosystem in cancer care consists of inclusive communities, data, tools, and resources.

#### 2.1.1 Our Communities

Our communities include providers or users of cancer care (healthcare providers, researchers, policy makers, lived experience co‐researchers, patients, caregivers, etc.). In STRONG‐AYA, AYAs with a lived experience of cancer co‐designed our work, to ensure its relevance, usability, and direct benefit for themselves and other patients [24, 25]. Our securely protected and purposefully defined personal data is underpinned by an ethical, secure, privacy‐ and confidentiality‐preserving technology platform.

### 2.2 Strong‐AYA as a Learning Healthcare System

The digital tools and resources developed within the STRONG‐AYA initiative collectively form a distinct Rapidly Learning Healthcare System by cresting and enabling culture and resource where stakeholders (including young people, clinicians, researchers and service providers) across borders are co‐operating, contributing, can view appropriate information and personally relevant outputs from the analyzed data, interact with research insights and communities, identify and act upon clinically relevant and user‐centered questions in real time.^1^ ^,^ ^2^ This is being applied to enable continuous improvement in AYA cancer care through cycles of inclusive discovery and implementation, starting with consensus priority areas for improvement—Cancer survival rates and Mental Health outcomes. Our system is designed with scientific enquiry, informatics, incentives, and an improvement culture aligned. This is catalyzing continuous cyclical improvement and innovation, using best practices within our collaborative, data science, and information technology methods. We have always embedded patients and their supporters as active (and driving) participants across our elements, as we integrate the medical and healthcare data with new and existing user experience (patient‐reported but non‐identifiable) data which is generated within the context of their treatment and experiences to identify and act upon priority areas for improvement [26, 27, 28].

Our data ecosystem is organized on three tiers: local, partial national (or regional) structures, and national systems—defined by the number of institutions and extent of data collected.

Local systems are narrow by design, but benefiting from granular and contextualized details—namely, they maintain and manage local data (e.g., in one specific hospital). For example, STRONG‐AYA in Poland consists of one participating hospital with their electronic patient data, and in Italy we include one providers' AYA cancer dataset.

Partial national (or regional) structures are broader, seeking shared learning across at least two locations. The Yorkshire Specialist Register of Cancer in Children and Young People (YSRCCYP)—for example—collects and harmonizes data from 3 comprehensive cancer centers and 10 local cancer treatment environments [29] including an entire population of around 5.4 million, contributing to regional public health and partially generalizable to other regions.

By contrast, national structures are even broader allowing for insights generalizable to a whole country. In the Netherlands, the national AYA research infrastructure can produce insights on incidence, risk factors and mechanisms behind medical and psychosocial effects experienced by patients. Examples of products resulting from this infrastructure are longitudinal observations on AYAs' PROs around diagnosis and followed up several years later (*COMPRAYA*) as well as cross‐sectional observations in AYAs who are > 5 years post‐diagnosis [30] [31]. Patients' clinical information (e.g., site, stage, detailed treatment, recurrence(s), toxicity and comorbidity) is linked from the Netherlands Cancer Register to allow for contextual and group‐level insights into the reported outcomes. Anonymized Data is freely available for other researchers upon approved application. France has two hospitals serving their local city and beyond, each providing regional‐based data collection in STRONG‐AYA currently but are working together to lay the groundwork for wholly national coverage. Two data ecosystems in France use local and proprietary data collection platforms respectively for PROs, allowing patients to take an active role in their own care.

Therefore, local and partially national ecosystems enable local champions to address local implementation, co‐own shared analyses of local data, and implement research findings and conclusions locally. By integrating the local and partially national data ecosystems within STRONG‐AYA we ensure the availability of a robust Learning Healthcare data ecosystem infrastructure across regions and nations which will allow additional local service providers to add their own ecosystem to this endeavor (see Figure 1 below).

**FIGURE 1:**
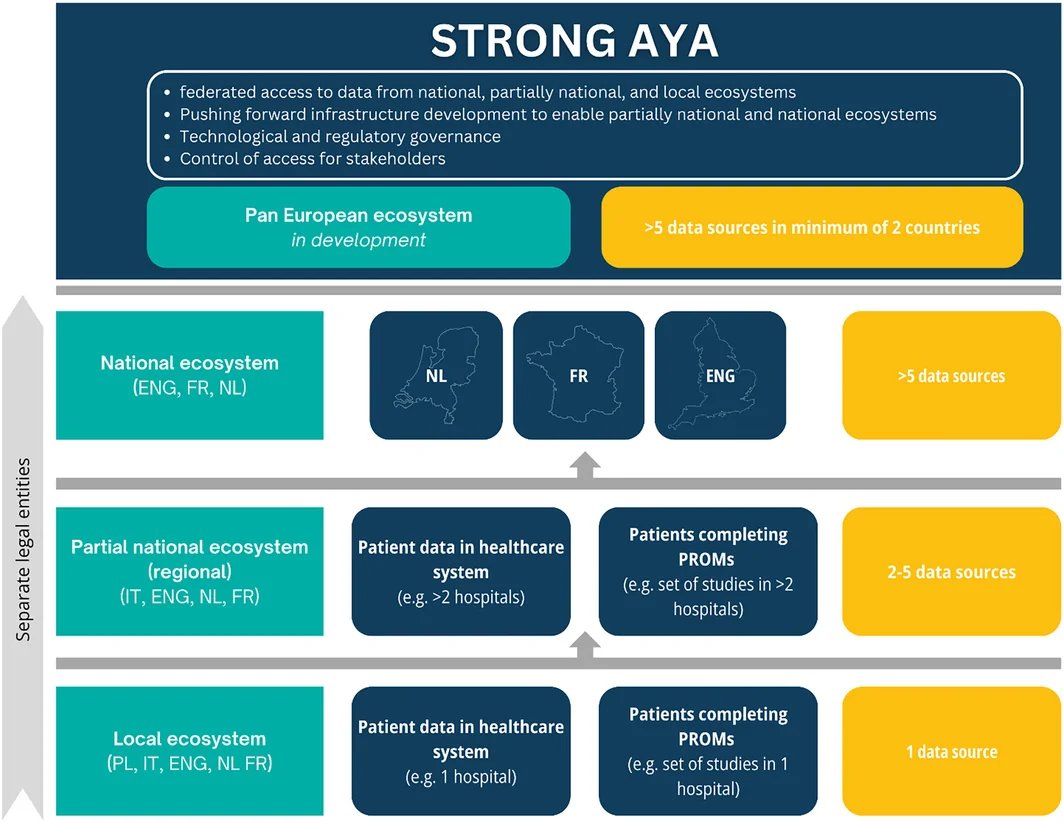
Structure and interplay of local, national, and multinational European data ecosystems in STRONG AYA.

### 2.3 Data Acquisition, Storage and Analysis That Provides Full Protection of Confidentiality

The technical implementation of the STRONG‐AYA initiative including its research and resulting products (including publications, statistics, software, etc.) abides by the principle of accessibility. That means that any outputs are made available and understandable by all stakeholders involved, with the exception of patient‐level data which remains fully private and protected inside institutional data repositories, in line with the privacy‐and confidentiality‐protection principles.

By using a well‐tested Federated Learning software infrastructure, researchers can perform data analysis without exchanging individual patient‐level data. Instead of combining all the patient data into a single data repository, STRONG AYA partners run the analysis on their own data and report only institutional cohort‐based results back to a central computer, which combines each partner's independent analysis result into a single common global result. The federated learning method is one instance out of a broader suite of privacy‐enhancement technologies (PETs); specifically, federated learning uses a “privacy by design” principle, since institutional results are shared and combined, but individual patient‐level data itself is never revealed.

While the STRONG‐AYA consortium adheres to the Findable‐Accessible‐Interoperable‐Reusable (“FAIR”) data management principles [32], this *does NOT imply that data is openly accessible* instead ensuring suitable data for analysis using “metadata” annotations describing the local data coding allowing computer code (remotely, centrally) to interact accurately with the data. No one single coding structure takes precedence over any other. OMOP (Observational Medical Outcomes Partnership) Common Data Model (“CDM”) is popular, but any member's data coding can be included without recoding. A “metadata overlay” specifying the local coding dictionaries enables data harmonization during analysis, creating syntactic and semantic interoperability that matures during the Consortium. In future STRONG‐AYA will enhance the dataset, by revising the metadata, not the data itself.

Railways are a simplified analogy for federated learning, with our own version of stations, trains and tracks. Local institutions become “stations” where patient‐level data safely resides. Though never shared externally, the patient data are, at the very least, pseudonymized (e.g., replacing a medical record number with a generated research‐only identifier) or fully anonymized, according to the local rules and regulations of the institution. In keeping with data minimization principles, birth dates and postcodes are automatically removed after extraction from source, and all dates are replaced by time intervals from a reference event (such as diagnosis). Local researchers within some institutions (e.g., a hospital serving one region) may retain separate awareness (“keys” or similar linkages as defined within its' own formal governance permissions) of participant identities, although these cannot be re‐accessed from within the data station. This can enable variables (such as partial postcode) to be analyzed locally where it adds value; in considerations of socio‐economic deprivation, for example. Together with our standard “COS,” mappings with universal lexica, ontologies and dictionaries (such as National Cancer Institute Thesaurus, NCIT) are inserted as *metadata*. The enriched metadata conforms to Findable‐Accessible‐Interoperable‐Reusable (FAIR) data management principles. As FAIR data, these data stations are not only usable to human operators, but the data can also be inter‐operably and dynamically processed by automated analytical software.

Next, our analogy requires “trains”; these represent the statistical analysis software. The research question or statistical analysis needs to be written as software code, and then exact copies of the code are sent out to each station. When a train arrives at a particular station, it analyses only the FAIR‐ified data placed inside that station; no other data can be processed or accessed by this train. At the end of its processing, the single institution cohort‐based result is reported by this train. As additional safety guardrails, STRONG AYA requires that all such analytic code be open sourced, independently validated and checked against malware content, and will only process results if a minimum sample size constraint is met within a station (e.g., *n* > 10).

Lastly, our analogy needs “tracks” which serve several essential cybersecurity functions. First, secure web industry‐grade encrypted telecommunication channels are needed, so that each train can report its analytical results back to a central computing device. Second, the central computer collects the institutional result of all the trains—crucially, not any of the individual patient data—and combines it into a global result for the entire consortium potentially providing the analytic detail of individual patient meta‐analysis without the sharing of any individual patient data. Third, the trains themselves have to be uniquely tagged and continuously monitored, so that only permitted analyses are running within the consortium. STRONG AYA uses the independently cybersecurity‐audited Vantage6 software infrastructure as “tracks,” which is hosted by an independent technical support fee‐for‐service contractor that has neither intellectual property nor data accessibility rights within the consortium.

### 2.4 Ethical and Legal Aspects of Federated Learning

STRONG‐AYA chose this federated data ecosystem to promote autonomy, solidarity, openness, diversity and choice among its participating members. This autonomy means that local ecosystems and national ecosystems always fully control their respective data. Our solidarity means AYA altruistically gave consent for their personal healthcare data to improve future healthcare provision. As patient‐level data never leaves its source, malicious actors—inside the consortium or from outside—would not be able to re‐identify data, even if they were able to decrypt the telecommunications channels. Our open methods require analyses to be protocolized a priori and easily auditable as open access computer code stored on a persistent software version control platform (such as GitHub). Our diversity brings inclusivity across heterogeneous data, providing a means to answer important clinical questions for rarer health conditions, as exemplified by AYAs diagnosed with cancer. As control of the data is pursued at a local level, the individual choice to opt in or out of specific analyses can be met through ongoing patient engagement, without requiring modification to the centralized database.

Legally, our federated data ecosystems have put in place explicit “Consortium” agreements, Data protection impact assessments (DPIAs), Data access framework agreements, and Infrastructure service agreement(s) with a technology support service provider(s) that host the technical and computational components, that have no say whatsoever in research questions, data or insights. The legal construction of STRONG AYA follows from open access examples (see https://www.medicaldataworks.nl/governance, and www.strongaya.eu) where we provide redacted specific documentation from our consortium once finalized.

All analyses produce insights (e.g., descriptions of the mental health challenges of the participants) collected as a group or subgroup, which are then permissible to be disseminated and shared. However, the granularity of insights accessed depends on the type of stakeholder (Table 1). To prioritize data security, all stakeholder systems need to specify some access and control protocols to block certain information reaching specific stakeholders. These insights are available in most detail for those with the highest level of access (e.g., senior researchers within the consortium with explicit consortium permissions to view that level of detail) who may be able to see details of the analytic code itself, the approach, and statistical results such as regression coefficients and residuals. Others, such as researchers external to the consortium or healthcare providers, with lower levels of approval may see the insights described and summaries of statistical findings. This can be presented in a format and manner in keeping with their training in the field, which may differ for example between some experts by lived experience within the consortium seeking to understand their own situation in context, and some more junior researchers seeking to identify supportive data for a research grant application.

**TABLE 1:** Levels of access to technical and analytic elements of the consortium data.

Access level	Activity
International Administrative	View and edit rights to the coded technical architecture of the international data ecosystem. This access is reserved to those who manage and maintain the data ecosystem, but those individuals cannot post use cases or run analyses.
Local administrative	View and edit rights to an institutional data station. This user access is for local technical staff who can generate the necessary authentication keys of their data station, enable appropriate end‐to‐end encryption (i.e., data station‐to‐backend), and so require individual level access to the local data station for maintenance purposes. They will also not post use cases, epidemiological questions or statistical analyses.
Full access	Complete view rights of content but without ability to edit. This level will be reserved for core and enduring consortium members and their teams within institutional member organizations. They will be able to look up descriptive statistics, post new use cases, pose epidemiological questions and formulate statistical hypotheses for validation by the consortium, which can be answered with the available data via the user interface. They will NOT have access to individual data stations nor can they create data analytic queries which have not yet been validated by the consortium members.
Partial access	View rights relevant to approved stakeholders who are not core consortium members and new members. These members will have restricted access to descriptive statistics. For example, a commercial partner might be allowed restricted access to descriptive statistics but not to deeper epidemiological modeling. This would be an access level applicable to some cancer service users as well. This will be subject to bona fides assessments, data protection and Infrastructure user agreements. For example a patient who has given consent to take part in a specific study to address a use case, or researcher in a specific institution with an institutional profile compatible with their involvement, may access more detail of analyses of specified use cases only. For example, pharmaceutical companies may not be permitted to develop queries/models involving drugs administered or procedures carried out without that request being considered by the Consortium as a whole. Data that is used for any such analyses may also be limited according to national ethical and regulatory frameworks. For instance, data on ethnicity cannot be utilized by law in France, so analyses of it may not be acceptable to be viewed by French participants.
Observational access	View rights to insights produced by the Consortium, but without access to analysis results themselves is provided for the wider public, without access to deeper online interfaces. What the public is allowed to view will be pre‐agreed by the Consortium members and is subject to local approvals.

These access levels are not static. External users of the system (e.g., healthcare managers in a specific hospital) can request access to a different insights access level if they undertake the consortium membership process and this level of access is commensurate to their role and objectives. Therefore (in future) a medical devices commercial partner may not be permitted to develop analyses involving specific special procedures carried out; however, such information might be made accessible to clinicians and researchers who wish to develop prediction models.

For the purpose of implementation in healthcare service development and provision, results and epidemiological models will be presented in a cognitively accessible way. To meet various stakeholders' communication needs, we developed a suite of user‐friendly data visualization web‐browser based “dashboards.” A web‐browser based application partly decouples the communication/dissemination aspects from using the underlying and complex infrastructure technology. Dashboards are being *co‐designed and co‐created* by clinicians, researchers, AYAs and policy makers. Different individuals will often see different dashboards. We plan to integrate AYA‐specific research questions, such as EuroCare enquiries, patient‐driven questions (from discussion fora) and policy requests (such as by the European Cancer Organization).

Analyses can be scheduled (time‐based), ad hoc (e.g., a specific one‐off clinical hypothesis) or on demand with appropriate consortium permission (e.g., a user wishes to see an update). Our data security therefore considers statistical analyses and re‐analyses. Questions such as the numbers of persons “diagnosis positive” (or negative) could risk security if only very few patients have had data added to the system between analyses. Our infrastructure is designed, therefore, to block analyses that do not have a default of 10 cases or cases added since last analysis.

Data insights with appropriate characteristics can however be accessible “in real time.” For example, clinical services and healthcare professionals working within the consortium membership (e.g., a hospital AYA clinic in France, caring for AYA who have consented to the consortium data collection and contributing data to a French data station) will only see appropriately high‐level insights, but can see specific and clinically useful insights gained from the data analysis when consulting with an individual participating AYA. So, for example, the self‐report mental health data for an individual patient may include a “reference range” to provide context and aid in clinical interpretation and case selection for clinical escalation. Due to our initiative that reference range does not need to be fixed—it dynamically updates based upon real‐time analysis of the datasets in the federated data system. And that reference range can be selected to indicate the interquartile range for a relevant aspect of that patient's care –such as their age‐group (15–24, 24–30 etc.), or specific cancer type (e.g., bowel carcinoma), or a specified time since cancer diagnosis (patients between 2 and 3 years from cancer diagnosis). Therefore, the reference range for a parameter becomes dynamic, regularly updated as new data is added, and (when useful) can reflect an aspect of interest to the patient and clinician for that consultation. However, privacy in federated learning remains underpinned by the unfeasibility of reverse‐engineering a participants' identity from analysis results (just as individual identity cannot be reverse‐engineered from analysis results in most research publications).

### 2.5 Governance

STRONG‐AYA has chosen a governance model which includes expected and possible incentives and motives of ecosystem partners, access to our findings, day‐to‐day operations, and compliance with laws and standards. The model describes:

1. Founding principles: to guide future decision‐making.
2. Legal and ethical frameworks for each partner; informing participants, promoting intellectual property sharing and democratizing benefits.
3. Members' rights and responsibilities.
4. Data analytic and technical standards, such as data security, integrity, privacy, and access rules.

All consortium members are expected to be transparent in their incentives and motives. Individuals have one or several roles in a dynamic system over time, as proposed by Oliveira et al. [33] (Figure 2).

**FIGURE 2:**
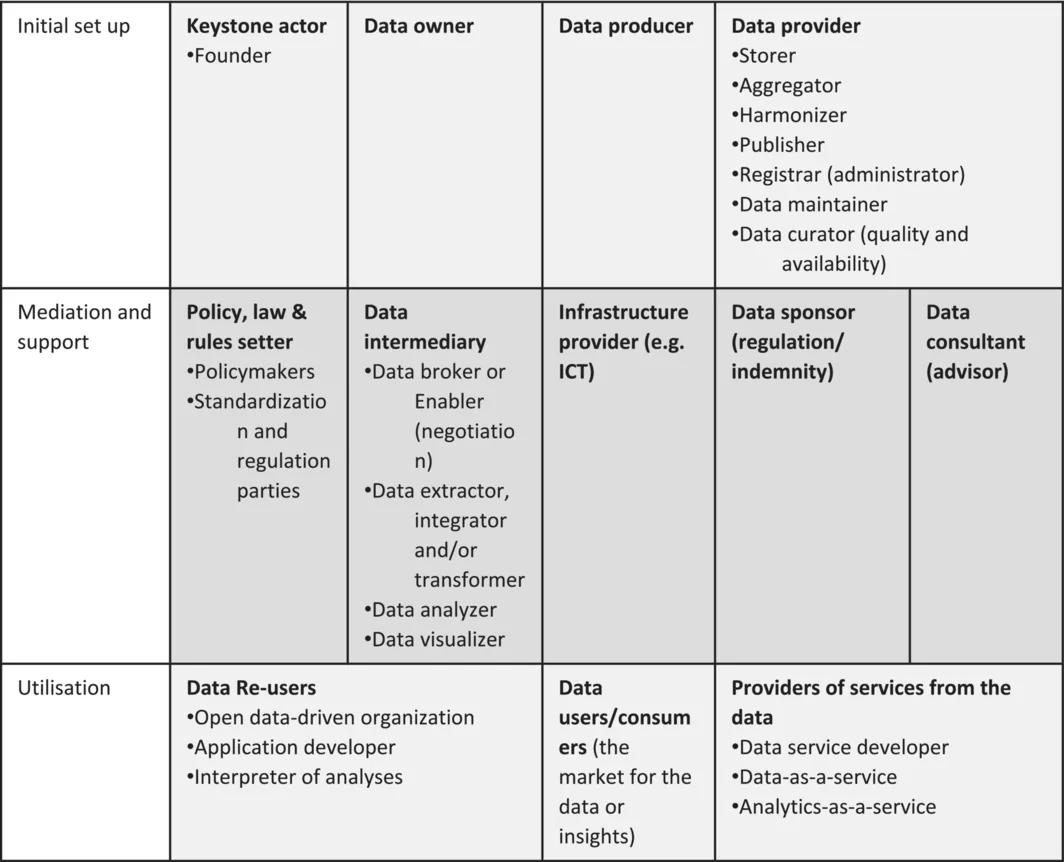
A broad taxonomy of potential roles within a data ecosystem. *Source:* Adapted from [33].

The core incentives and motives for STRONG‐AYA Consortium membership are summarized as:

1. Improvement in the provision of healthcare services.
2. Performing ethical research on large datasets.
3. Improving patient outcomes.

Different members may have or develop other incentives for participating, such as discovery (mining data in rare disease), accelerating implementation (sharing administrative workload within diverse datasets), or sustaining collaborative clinical practices.

We have chosen an inclusive, ethical, patient‐centered ethos of “nothing about me without me,” moving beyond traditional patient and public engagement to be founded on the interests, preferences, and values of patients in the Consortium. To do so, we sought the guidance of a Patient Advisory Board (PAB) of young people with lived cancer experience, which embeds Equity Diversity and Inclusion across dimensions such as expertise, geography, and tumor type. The PAB was formed initially from members of the Youth Cancer Europe organization (Youth Changing Europe—Youth Cancer Europe) currently extending to other member nations and centers. This supports STRONG‐AYA by sharing knowledge, facilitating feedback to patient communities, and wider (e.g., political) outreach. This includes co‐creation of the patient platform, data visualizations, tutorials, and warning or explanatory messages.

Importantly, our tools and actions aim to complement and optimize face‐to‐face healthcare, so patients can easily and reliably exercise their right to access/retrieve their own STRONG‐AYA health data (through their own or hospital systems). By utilizing the patient‐facing dashboard, they can access and better understand their own health data compared to other AYAs and adjust access to information on the purposes and uses of their data for specific use cases. This provides a voice in designing our tools and insights into the accessibility of formats in which data is presented, for instance in cases of differing levels of digital or healthcare literacy. Training processes can support users of our tools, embedded in routine care, to enhance human communication and support personalized shared decision‐making between AYA and health care providers.

### 2.6 Open and Transparent Science

STRONG‐AYA abides by the principle that our research and insights (including publications, statistics, software, etc.) shall be accessible to all stakeholders. Analytic algorithms are openly accessible and constantly reviewable/critiqued. All publications are open access; dissemination activities are openly available.

STRONG‐AYA governance remains inclusive of all local policy and legal agreements for each partner, and at pan‐European levels. Examples include European Ethical Principles for Digital Health (2022), and General Data Protection Regulation (GDPR; https://gdpr‐info.eu/), and applicable sub‐legislations relating to purpose limitation; data minimization; accuracy; storage limitation, integrity, confidentiality; and accountability. STRONG‐AYA has chosen to practice explicit data anonymization for analysis or re‐analysis, to protect against unintended re‐identification of individual data subjects (e.g., cohorts > 10 as default, adjusted with justification).

All prospective participants provide and document informed consent for their data to be used within the ecosystem. Retrospective (historical) data must meet all three grounds of GDPR article 4: (a) participant offered freely given, specific, and informed consent (Article 6 (1) (a)). (b) processing requires a legitimate interest of controllers AND that legitimate interest cannot reasonably be achieved by alternative means AND the interests and fundamental rights and freedom of the participant do not take precedence over the legitimate interests of the controller(s); (c) the data is anonymized and therefore falls outside GDPR. An example is the legitimate interest of testing hypotheses for public health.

A Committee for Coordination (CfC), established by NKI, of local ecosystem members including patient advocates, oversees our pan‐European ecosystem, critically ensuring our principles are adhered to. The CfC also ensures rules are defined for the submission of use cases (analyses of the dataset for a purpose), and analyses that follow. This can mean disputes and ambiguous situations are rapidly addressed, and more successes, for example, intellectual property, can be shared.

Our approach was informed by several other parallel initiatives. We are contributing actively to the legally binding implementation of the European Health Data Spaces (EHDS) and can compare our approach in STRONG AYA with others such as: FeatureCloud, which uses encryption and blockchain‐based computations and DIGIONE (DigiOne—I3), which pioneers an EU‐wide rapidly learning oncology system aiming to optimize cancer treatment selection. Other similar significant initiatives which we considered are The Health Outcomes Observatory (H2O) project (Health Outcomes Observatory—Health Outcomes Observatory) which aims to build an international European ecosystem to incorporate wide health outcomes into decision making across several diseases, with a federated data method and digital tools for patients. H20 is creating separate not‐for‐profit legal entities in each country, to facilitate further grant acquisition, requiring significant bureaucracy.

We were also informed by Blueberry, a European Reference Network (ERN) project, aiming to overcome challenges to a pan‐European sarcoma registry, using a federated data infrastructure to connect France, Italy, Austria, Norway and Spain. Key challenges included governance, GDPR, long‐term financial investment for low‐prevalence cancers and data harmonization (EURACAN—BlueBerry successfully completed and delivers final Blueprint).

The difference between STRONG‐AYA and these other initiatives is its simplified governance infrastructure, principles of operation, and critically the federated learning technological infrastructure deployed at local and national levels. We believe we have learned that by defining our ecosystems by data source and technical functionality we may undertake earlier analysis on a larger amount of data.

STRONG‐AYA have chosen to adhere to the Findable‐Accessible‐Interoperable‐Reusable (“FAIR”) data management principles [33]. However, all STRONG‐AYA analysis code is reviewed by local data experts (being publicly available) to cross‐verify its mathematical function and lack of malicious action. Then locked code is packaged into standalone, independent, and operating‐system agnostic software applications (or “Docker containers” https://www.ibm.com/think/topics/docker) which we refer to as “trains.” One train may contain a generalized regression model, and another statistical summaries of cancer prevalences. Each train addresses a specific use case, including planned analysis, applications and foreseen insights.

Our chosen approach requires consortium‐wide shared technical support, knowledge sharing, and deployment of customizable software tools. In contrast, data centralization designs send data to a principal investigator and their computing and human resources clean and analyze the data. Our federated approach requires expertise to remain dispersed among the partners, which we view as a positive, democratizing collaboration, embedding a willingness among institutions to share expertise and the maturation of research capacity, rather than keeping these in one location.

Blueberry was a European Reference Network (ERN) project, aiming to overcome challenges to a pan‐European sarcoma registry, using a federated data infrastructure to connect France, Italy, Austria, Norway, and Spain. Key challenges include governance, GDPR, long‐term financial investment for low‐prevalence cancers and data harmonization (EURACAN—BlueBerry successfully completed and delivers final Blueprint).

STRONG‐AYA is its focus on being a pan‐European initiative with an already established multi‐national scope, led by active champions within global AYA cancer research and policy, collaborates with international NGO, professional and patient advocacy organizations, and it seeks to address international issues such as cross‐border research. The multi‐national element emerges during the integration of local and national data ecosystems which function on the same infrastructure and governance systems but embed and adapt these within current working practices. Until very recently, North American initiatives in learning from data across organizations in AYA with cancer have largely relied upon traditional data pooling. Examples include the Data for the Common Good initiative (Connect. Share. Discover.—Data for the Common Good, a Childrens Oncology Group‐supported organization) where several of our consortium members are also active, working within the confines of diverse intercontinental ethical and governance regulations, and the need for data export to a central controller and consequent partial loss of data ownership by the local data collector. Over time, the field may see the opportunities offered by federation to enable AYA cancer outcomes research at an intercontinental scale, as already done in other cancer areas [34]. Since the governance, technical, and operational infrastructure of the data ecosystems are in place across European countries, funding is being sought to extend our model across other continents, including to N American AYA cancer data.

## 3 Current Status

The STRONG‐AYA Consortium currently has data on 29, 482 individual AYA living with and after cancer, from our five European nations. This includes whole population data from the Netherlands, regional population‐based data and data from two reference centers in the UK, data from two national reference centers in France, and single national reference center data from Italy and Poland. Use cases for the dynamic modeling of mental health and survival probability are agreed and healthcare and patient dashboard exemplars for these are in advanced development.

## 4 Conclusions and Key Challenges

The STRONG‐AYA Consortium demonstrates the value of collaborative work across multiple stakeholder groups with a common goal in developing and sustaining Learning Healthcare Systems. The large‐scale, better powered new outcome insights have direct applications in science, macro‐level service development, meso‐level service implementation, and micro‐level clinical care delivery for the improvement of patient outcomes.

Enhancing the huge value of traditional registries, federated learning can optimize access to larger and more impactful results. STRONG‐ AYA has chosen a solidarity‐based data governance approach that assesses the overall public value likely to derive from each data use and its risks, using https://pluto.univie.ac.at/. This extends beyond “personal” data to protect, and anonymized data to use freely and defines: (1) creating data most likely to bring meaningful public value without creating undue risk. (2) Assessing then preventing or mitigating harm from data use. (3) Ensuring that a fair portion of the benefits from data use are redistributed to each who made it possible [34, 35, 36]. Our choice to use Application Programming Interfaces “APIs” is accepting of the inevitable new software implemented in healthcare centers.

All stakeholders seek to continue ensuring no data leaks “at Source”, “in Transit” or from pooled analyses which are as relevant to federated systems as to traditional methods of sharing and analyzing pooled data across borders. ‐ transit and centralized data leaks are reduced. Our sequential cybersecurity audits demonstrate no current method to penetrate our ecosystem or analytic transit pathways from outside.

Despite these opportunities, similar to previous initiatives, we do foresee challenges in scalability. While there are several benefits in maintaining local autonomy and control over assets as outlined above, some scalability challenges arise precisely from the openness and need for dynamic adaptation to local ethical codes and evolving healthcare and technical infrastructures at local levels. Lastly, the identification of financial resources to sustain the ecosystem components described above (the community with its expertise and technical infrastructure) will ensure the ongoing functioning of our initiative. To ensure its continuity we have set criteria for new members: local technical and legal expertise, data and metadata, which applies to all those interested in joining the Strong‐AYA ecosystem:

- *Provision and availability of retrospective data: Is data available and can it be analyzed?* According to which local GDPR and local legal/ethical approaches.
- *Data collection and consent norms for prospective data: How is data collected?* According to which local Ethics Board approvals.
- *Operational norms and standards: How does each institution operationalize the Consortium internally?* According to which local cultural and data governance regulations.
- *What are your technical standards (i.e., guarantee data security, integrity, and privacy)?* According to which local developments technical and digital governance regulations.
- *What are the legal bases for consenting, collecting, and using patient data in research in each country and institution?* According to which local interpretation of legal frameworks and which established practices.

Therefore, to ensure its sustainability over time, STRONG‐AYA prioritizes patients' guidance and access to their own data, transparency in data usage, and most of all enhancing the positive impact patients experience as a result of their data being utilized through improved healthcare provision. Similarly, ensuring stakeholder acceptability in the type and format of presenting insights, as well as focusing use cases on what is relevant and useful to them will likely enhance its uptake. This added value needs to be communicated to patients at recruitment followed by patients being able to view in real time how their data can be used for meaningful conversations. Healthcare professionals also ask for the insights to be useful, comprehensible and acceptable. This requires integration of insights within local systems, and value arising from access to our portal even embedded within Electronic Health Records. While federation could be perceived as loss of primacy over collected datasets, data cannot be utilized unless there is an a priori collaboratively agreed analysis algorithms, ensuring data contributors would be actively involved in how the data is utilized.

Resources are, unsurprisingly, a challenge, requiring income, in rarer illnesses, where data is not always viewed as a good investment for commerce, industry or governments prioritizing aging populations. Future solutions include diversifying a portfolio of funding, seeking utility of analyses to governmental or policymakers, and ultimately an attractive enough offer to implement a subscription service for some users, such as is used by the UK Cancer Registration Service, charging its costs for academic data exports. At the heart of this are the young people with lived experience of cancer.

The scaling and sustaining meaningful patient and public involvement, particularly embedding patients' voice in the continuously improving ecosystem infrastructure and content, remains a challenge. One such initiative for ongoing co‐design is YARN (https://youthcancereurope.org/yarn/). This can keep STRONG‐AYA positioned to empower, with personalized tailored information, opportunities to explore their own data and enhanced digital and health literacy. Seamless real‐time integration of research insights into routine clinical care benefits individuals, society, and clinical communities.

Our experience with this collaboration template is successful so far, pending successful multicenter implementation, and provides an improvement design to further solve several current problems of AYA with cancer and an infrastructure for future cycles of inclusive discovery and continuous improvement including system evaluations. The nature of data that can be collected, managed and analyzed in this manner is increasing over time. Genetic, genomic, radiological, histopathological and many more categories of digital data are increasingly defined and may require integration in due course. Initiatives in several of these domains are underway within our consortium for AYA with cancer, in the Netherlands and UK nationally, and in very recently funded wider research collaborations between all our groups and global collaborators, where specific analyses will examine the effects of multiple environmental exposures on causes and outcomes of cancer in AYA (https://tinyurl.com/MIREYA‐Cancer) and screening protocols for late effects of AYA‐onset cancer (confidential communication Sept 2026), respectively.

By embedding age‐appropriate co‐created PROs in dashboards visible to service users and their healthcare service providers, we are providing a privacy‐preserving multi‐center solution for evidence‐based conversations and decision making in routine healthcare. The Strong‐AYA Learning Healthcare System for AYA‐onset cancer can empower discovery by health researchers, digital innovation, and innovative insights for clinicians, managers and policymakers who aim for transformational improvements in the quality and outcomes of current clinical practice through the development of a Learning Healthcare System in AYA‐onset cancer care [37].

## Funding

STRONG AYA is funded by the European Union under grant agreement no 101057482. Views and opinions expressed are however those of the author(s) only and do not necessarily reflect those of the European Union or the Health and Digital Executive Agency (HADEA). Neither the European Union nor the granting authority can be held responsible for them. STRONG AYA is also funded within the UK by Innovate UK under grants 10038931, 10039273, 10041045, and 10044189. Views and opinions expressed are however those of the author(s) only and do not necessarily reflect those of UK Research and Innovation and Innovate UK. Neither UKRI nor the granting authority can be held responsible for them.

## Conflicts of Interest

The authors declare no conflicts of interest.

## Data Availability

The data that support the findings of this study are available on request from the corresponding author. The data are not publicly available due to privacy or ethical restrictions.

## Endnotes

^1^
What should a learning health system look like?|BMJ Open Quality accessed August 2026.

^2^
Learning Health System Series—NAM Accessed August 2026.

## References

[1] Group AaYAOPR , Closing the Gap: Research and Care Imperaives for Adolescents and Young Adults With Cancer (National Institute of Health, National Cancer Institute, and Livestrong Young Ault Alliance, 2006).

[2] A. G. Close , A. Dreyzin , K. D. Miller , B. K. N. Seynnaeve , and L. B. Rapkin , “Adolescent and Young Adult Oncology‐Past, Present, and Future,” CA: A Cancer Journal for Clinicians 69 (2019): 485–496, 10.3322/caac.21585. 31594027

[3] S. Gupta , A. Harper , Y. Ruan , et al., “International Trends in the Incidence of Cancer Among Adolescents and Young Adults,” Journal of the National Cancer Institute 112 (2020): 1105–1117, 10.1093/jnci/djaa007. PMC7669231 32016323

[4] D. R. Lewis , N. L. Seibel , A. W. Smith , and M. R. Stedman , “Adolescent and Young Adult Cancer Survival,” Journal of the National Cancer Institute Monographs 2014, no. 49 (2014): 228–235, 10.1093/jncimonographs/lgu019. PMC4841167 25417236

[5] A. Trama , L. Botta , R. Foschi , et al., “Survival of European Adolescents and Young Adults Diagnosed With Cancer in 2000‐07: Population‐Based Data From EUROCARE‐5,” Lancet Oncology 17 (2016): 896–906, 10.1016/S1470-2045(16)00162-5. 27237614

[6] S. C. Adams , J. Herman , I. C. Lega , et al., “Young Adult Cancer Survivorship: Recommendations for Patient Follow‐Up, Exercise Therapy, and Research,” JNCI Cancer Spectrum 5 (2021): pkaa099, 10.1093/jncics/pkaa099. PMC7919337 33681702

[7] C. Chao , S. Bhatia , L. Xu , et al., “Chronic Comorbidities Among Survivors of Adolescent and Young Adult Cancer,” Journal of Clinical Oncology 38 (2020): 3161–3174, 10.1200/JCO.20.00722. PMC7499612 32673152

[8] J. D. Palmer , M. D. Hall , A. Mahajan , A. C. Paulino , S. Wolden , and L. S. Constine , “Radiotherapy and Late Effects,” Pediatric Clinics of North America 67 (2020): 1051–1067, 10.1016/j.pcl.2020.08.001. 33131534

[9] C. Ryder‐Burbidge , R. L. Diaz , R. D. Barr , et al., “The Burden of Late Effects and Related Risk Factors in Adolescent and Young Adult Cancer Survivors: A Scoping Review,” Cancers 13 (2021): 4870, 10.3390/cancers13194870. PMC8508204 34638350

[10] E. Woodward , M. Jessop , A. Glaser , and D. Stark , “Late Effects in Survivors of Teenage and Young Adult Cancer: Does Age Matter?,” Annals of Oncology 22 (2011): 2561–2568, 10.1093/annonc/mdr044. 21427066

[11] M. M. Fidler , C. Frobisher , M. M. Hawkins , and P. C. Nathan , “Challenges and Opportunities in the Care of Survivors of Adolescent and Young Adult Cancers,” Pediatric Blood & Cancer 66 (2019): e27668, 10.1002/pbc.27668. 30815985

[12] G. K. Perez , J. M. Salsman , K. Fladeboe , A. C. Kirchhoff , E. R. Park , and A. R. Rosenberg , “Taboo Topics in Adolescent and Young Adult Oncology: Strategies for Managing Challenging but Important Conversations Central to Adolescent and Young Adult Cancer Survivorship,” American Society of Clinical Oncology Educational Book 40 (2020): 1–15, 10.1200/EDBK_279787. PMC7328818 32324424

[13] A. Ferrari , D. Stark , F. A. Peccatori , et al., “Adolescents and Young Adults (AYA) With Cancer: A Position Paper From the AYA Working Group of the European Society for Medical Oncology (ESMO) and the European Society for Paediatric Oncology (SIOPE),” ESMO Open 6 (2021): 100096, 10.1016/j.esmoop.2021.100096. PMC8103533 33926710

[14] Collaborators GBDAYAC , “The Global Burden of Adolescent and Young Adult Cancer in 2019: A Systematic Analysis for the Global Burden of Disease Study 2019,” Lancet Oncology 23 (2022): 27–52. 10.1016/S1470-2045(21)00581-7 PMC8716339 34871551

[15] A. Bleyer , R. Barr , B. Hayes‐Lattin , D. Thomas , C. Ellis , and B. Anderson , “The Distinctive Biology of Cancer in Adolescents and Young Adults,” Nature Reviews Cancer 8 (2008): 288–298, 10.1038/nrc2349. 18354417

[16] R. M. Dommett , M. T. Redaniel , M. C. Stevens , M. C. G. Stevens , W. Hamilton , and R. M. Martin , “Features of Cancer in Teenagers and Young Adults in Primary Care: A Population‐Based Nested Case‐Control Study,” British Journal of Cancer 108 (2013): 2329–2333, 10.1038/bjc.2013.191. PMC3681013 23619924

[17] L. A. Fern , R. Birch , J. Whelan , et al., “Why Can't We Improve the Timeliness of Cancer Diagnosis in Children, Teenagers, and Young Adults?,” BMJ 347 (2013): f6493, 10.1136/bmj.f6493. 24170036

[18] M. R. Meeneghan and W. A. Wood , “Challenges for Cancer Care Delivery to Adolescents and Young Adults: Present and Future,” Acta Haematologica 132 (2014): 414–422, 10.1159/000360241. 25228567

[19] N. J. Levin , B. Zebrack , and S. W. Cole , “Psychosocial Issues for Adolescent and Young Adult Cancer Patients in a Global Context: A Forward‐Looking Approach,” Pediatric Blood & Cancer 66 (2019): e27789, 10.1002/pbc.27789. PMC10599653 31058418

[20] T. H. M. Keegan , R. Abrahao , and E. M. Alvarez , “Survival Trends Among Adolescents and Young Adults Diagnosed With Cancer in the United States: Comparisons With Children and Older Adults,” Journal of Clinical Oncology 42 (2024): 630–641, 10.1200/JCO.23.01367. PMC12040215 37883740

[21] A. W. Smith , N. L. Seibel , D. R. Lewis , et al., “Next Steps for Adolescent and Young Adult Oncology Workshop: An Update on Progress and Recommendations for the Future,” Cancer 122 (2016): 988–999, 10.1002/cncr.29870. PMC7521143 26849003

[22] Medicine Io , Digital Infrastructure for the Learning HealthSystem: The Foundation for Continuous Improvement in Health and Health Care: Workshop Series Summary., in Press; TNA (ed). Washington, DC, 2011. 22379651

[23] M. Das , “New Report From STRONG‐AYA on Addressing Needs of Adolescents and Young Adults With Cancer in Europe,” Lancet Oncology 25 (2024): 960, 10.1016/S1470-2045(24)00382-6. 38972327

[24] A. S. Darlington , K. Way , N. Collaco , et al., “Development of a Core Outcome Set for Adolescents and Young Adults With Cancer,” Lancet Oncology 27 (2026): e45–e54, 10.1016/S1470-2045(25)00533-9. 41449159

[25] U. Kosir , A. Totovina , D. Stark , et al., “Minimum Standards of Specialist Adolescent and Young Adult (AYA) Cancer Care Units: Recommendations and Implementation Roadmap,” ESMO Open 10 (2025): 105829. 10.1016/j.esmoop.2025.105829 PMC12514500 41033283

[26] A. Shah , A. K. Stewart , A. Kolacevski , D. Michels , and R. Miller , “Building a Rapid Learning Health Care System for Oncology: Why CancerLinQ Collects Identifiable Health Information to Achieve Its Vision,” Journal of Clinical Oncology 34 (2016): 756–763, 10.1200/JCO.2015.65.0598. 26755519

[27] P. Lambin , E. Roelofs , B. Reymen , et al., “Rapid Learning Health Care in Oncology: An Approach Towards Decision Support Systems Enabling Customised Radiotherapy,” Radiotherapy and Oncology 109 (2013): 159–164, 10.1016/j.radonc.2013.07.007. 23993399

[28] G. Price , R. Mackay , M. Aznar , et al., “Learning Healthcare Systems and Rapid Learning in Radiation Oncology: Where Are We and Where Are We Going?,” Radiotherapy and Oncology 164 (2021): 183–195, 10.1016/j.radonc.2021.09.030. 34619237

[29] K. J. Cromie , P. Crump , N. F. Hughes , et al., “Data Resource Profile: Yorkshire Specialist Register of Cancer in Children and Young People (Yorkshire Register),” International Journal of Epidemiology 52 (2023): e18–e26, 10.1093/ije/dyac195. PMC9908036 36228114

[30] O. Husson , M. J. L. Ligtenberg , L. V. van de Poll‐Franse , J. B. Prins , M. J. van den Bent , and M. C. van Eenbergen , “Comprehensive Assessment of Incidence, Risk Factors, and Mechanisms of Impaired Medical and Psychosocial Health Outcomes Among Adolescents and Young Adults With Cancer: Protocol of the Prospective Observational COMPRAYA Cohort Study,” Cancers 13 (2021): 2348, 10.3390/cancers13102348. PMC8152481 34068026

[31] C. Vlooswijk , L. V. Poll‐Franse , S. H. M. Janssen , et al., “Recruiting Adolescent and Young Adult Cancer Survivors for Patient‐Reported Outcome Research: Experiences and Sample Characteristics of the SURVAYA Study,” Current Oncology 29 (2022): 5407–5425, 10.3390/curroncol29080428. PMC9406992 36005166

[32] M. I. S Oliveira , G.d. F. Barros Lima , and B. Farias Lóscio , “Investigations Into Data Ecosystems: A Systematic Mapping Study,” Knowledge and Information Systems 61 (2019): 589–630.

[33] M. D. Wilkinson , M. Dumontier , I. J. Aalbersberg , et al., “The FAIR Guiding Principles for Scientific Data Management and Stewardship,” Scientific Data 3 (2016): 160018. 10.1038/sdata.2016.18 PMC4792175 26978244

[34] S. Theophanous , P. I. Lonne , A. Choudhury , et al., “An International Multi‐Centre Study to Develop and Validate Federated Learning‐Based Prognostic Models for Anal Cancer,” Nature Communications 17 (2026): 1–13. 10.1038/s41467-026-70297-3 PMC13133161 41832163

[35] B. Prainsack and S. El‐Sayed , “Beyond Individual Rights: How Data Solidarity Gives People Meaningful Control Over Data,” American Journal of Bioethics 23 (2023): 36–39, 10.1080/15265161.2023.2256267. 37879024

[36] B. Prainsack , S. El‐Sayed , N. Forgo , et al., “Data Solidarity: A Blueprint for Governing Health Futures,” Lancet Digital Health 4 (2022): e773–e774, 10.1016/S2589-7500(22)00189-3. 36307191

[37] P. P. Yu , W. S. Campbell , E. B. Durbin , L. N. Shulman , and J. L. Warner , “Twenty‐First Century Data Systems: Evolving Cancer Registries to a Learning Health System,” Learning Health Systems 10 (2026): e70080, 10.1002/lrh2.70080. PMC13078859 41988463

